# Community-acquired methicillin-resistant *Staphylococcus auereus* in a Malaysian tertiary centre in year 2009

**DOI:** 10.1186/1753-6561-5-S6-P18

**Published:** 2011-06-29

**Authors:** Z Zainol Rashid, N Bahari, A Othman, R Jaafar, NA Mohamed, I Jabbari, A Sulong, R Hashim, N Ahmad

**Affiliations:** 1 Dept of Medical Microbiology & Immunology, Faculty of Medicine,Universiti Kebangsaan, Malaysia; 2 Bacteriology Unit, Institute of Medical Research, Kuala Lumpur, Malaysia

## Introduction / objectives

Community-Acquired Methicillin-Resistant *Staphylococcus aureus* (CA-MRSA) is a pathogen recognized to be distinct from hospital-acquired MRSA phenotypically and genotypically. We aimed to identify CA-MRSA cases in UKMMC, their antibiotic susceptibility patterns and genotypic characteristics.

## Methods

Cases were identified prospectively from January to December 2009, where culture and antibiotic susceptibility results yielding pauciresistant MRSA isolates were suspected as CA-MRSA. The patients’ clinical data were collected and their specimens were sent for molecular confirmation and analysis at IMR.

## Results

Five cases of CA-MRSA were identified. The isolates had multisensitive pattern on antibiotic susceptibility testing and were resistant to only penicillin and oxacillin. All cases were skin and soft-tissue infections, namely diabetic foot with gangrene, infected scalp haematoma, philtrum abscess in a healthcare worker, thrombophlebitis complicated with abscess and infected bedsore. All five cases were confirmed MRSA by detection of *mecA* gene. SCC*mec* typing (*ccr* gene and *mec* complex gene) revealed SCC*mec* type IV for all except the infected bedsore case. Panton-Valentine Leucocidin gene was positive in all isolates.

## Conclusion

The clinical confines between methicillin-sensitive *Staphylococcus aureus*, CA-MRSA and “nosocomial CA-MRSA” are indistinct. Early recognition is necessary in instituting appropriate antibiotics and infection control measures. Continued surveillance of pauciresistant MRSA and molecular analysis is useful to identify emerging strains and their epidemiology and transmission, both in the community as well as healthcare settings.

## Disclosure of interest

None declared.

